# Supporting Informed Decision Making Online in 20 Minutes: An Observational Web-log Study of a PSA Test Decision Aid 

**DOI:** 10.2196/jmir.1307

**Published:** 2010-05-26

**Authors:** Natalie Joseph-Williams, Rhodri Evans, Adrian Edwards, Robert G Newcombe, Patricia Wright, Richard Grol, Glyn Elwyn

**Affiliations:** ^3^IQ Scientific Institute for Quality in HealthcareRadboud UniversityNijmegenNetherlands; ^2^School of PsychologyCardiff UniversityCardiffUnited Kingdom; ^1^Department of Primary Care and Public HealthSchool of MedicineCardiff UniversityCardiffUnited Kingdom

**Keywords:** decision aid, informed decision making, internet, prostate cancer, prostate specific antigen (PSA) test, user tracking, web-log, website transaction log file

## Abstract

**Background:**

Web-based decision aids are known to have an effect on knowledge, attitude, and behavior; important components of informed decision making. We know what decision aids achieve in randomized controlled trials (RCTs), but we still know very little about how they are used and how this relates to the informed decision making outcome measures.

**Objective:**

To examine men's use of an online decision aid for prostate cancer screening using website transaction log files (web-logs), and to examine associations between usage and components of informed decision making.

**Methods:**

We conducted an observational web-log analysis of users of an online decision aid, Prosdex. Men between 50 and 75 years of age were recruited for an associated RCT from 26 general practices across South Wales, United Kingdom. Men allocated to one arm of the RCT were included in the current study. Time and usage data were derived from website log files. Components of informed decision making were measured by an online questionnaire.

**Results:**

Available for analysis were 82 web-logs. Overall, there was large variation in the use of Prosdex. The mean total time spent on the site was 20 minutes. The mean number of pages accessed was 32 (SD 21) out of a possible 60 pages. Significant associations were found between increased usage and increased knowledge (Spearman rank correlation [ρ] = 0.69, *P* < .01), between increased usage and less favorable attitude towards PSA testing (ρ = -0.52, *P* < .01), and between increased usage and reduced intention to undergo PSA testing (ρ = -0.44, *P* < .01). A bimodal distribution identified two types of user: low access and high access users.

**Conclusions:**

Increased usage of Prosdex leads to more informed decision making, the key aim of the UK Prostate Cancer Risk Management Programme. However, developers realistically have roughly 20 minutes to provide useful information that will support informed decision making when the patient uses a web-based interface. Future decision aids need to be developed with this limitation in mind. We recommend that web-log analysis should be an integral part of online decision aid development and analysis.

**Trial Registration:**

ISRCTN48473735; http://www.controlled-trials.com/ISRCTN48473735 (Archived by WebCite at http://www.webcitation.org/5pqeF89tS)

## Introduction

Enabling patients to make informed decisions is the new benchmark for high quality care as recently highlighted by the NHS (National Health Service) Constitution document in which the patient’s right to choice and information were emphasized [1]. Decision aids have been developed to support patient participation in health care decisions [2]. Although decision aids are rapidly establishing themselves as the ideal medium for supporting informed decision making, we know very little about the best way to develop these interventions [3,4].

Decision aids are designed to facilitate informed decision making, which is characterized by improved knowledge and attitudes that are congruent with subsequent behaviors [5]. Since their introduction to clinical practice, research on decision aids has developed rapidly over the last fifteen years. Most research has focused on the effect of using decision aids on certain outcomes in a variety of clinical settings. Evaluations of decision aids, summarized in a Cochrane review [2], have shown that they increase knowledge and are more likely to lead to informed values-based decisions than routine care. We also know that users of decision aids tend to make conservative decisions or postpone interventions, especially when faced with surgical procedures: a trend which has significant implications for health service resource use [6,7].

Decision aids are therefore powerful tools that have the potential to influence patients’ health care decisions and use of services. Given their prominence in health care and policy making, clinicians and developers are realizing the need for a greater understanding of how decision aids are developed and used. If decision aids have the potential to influence patients’ decisions, then we need to know how we can optimize their design, particularly for diverse groups of users. We also need to know if usage of decision aids is sufficiently high to justify the expense involved in developing increasingly sophisticated decision support tools for patients.

Until recently, the formats of decision aids (eg, leaflets and videos) did not allow researchers to analyze patterns of use, particularly if the decision aid was used by the patient when alone. The migration of decision aids to the Internet, however, has offered the potential of analyzing use of decision aids in greater detail by using website transaction log files (web-logs). Within the context of website interface design, web-log analysis has long been realized as an important tool for examining website usability and usage [8-10]. Although researchers in other contexts have realized the practical use of web-logs for improving website interface design, developers of web-based decision aids have failed to recognize this potential. An early indication of the potential to use web-logs in the health care context was given by Molenaar [11], who examined the association between decision aid usage and several patient characteristics.

We believe, however, that far greater research opportunities are offered by analyzing patients’ usage of decision aids using web-logs. First, we can examine associations between usage and key outcomes that decision aids are known to affect, including the components of informed decision making. In the current study, we hypothesized that increased usage of the decision aid would be associated with informed decision making. Secondly, we can explore the usage data to inform the future design of web-based decision aids. Based on the field-testing of Prosdex in its development stage [12], we hypothesized that the users would not represent a homogeneous group, and that they would use the decision aid differently from one another.

Prosdex [13] is one of the first web-based decision aids developed to help men decide whether or not to have the prostate-specific antigen (PSA) test for prostate cancer. The decision is difficult due to the inaccuracy of the test and the fact that the natural history and management of prostate cancer is poorly understood. Controversy surrounding the test has increased recently with the publication of contrasting data: a European randomized controlled trial (RCT) showed that PSA screening reduced mortality by 20% [14], while results of a trial in the United States indicated no difference in mortality after 7 to 10 years follow-up [15]. Prosdex was subsequently developed with the mandate of promoting informed decision making, the strategic aim of the UK Prostate Cancer Risk Management Programme (PCRMP) [16]. We aimed to observe participants’ use of Prosdex using web-log data to identify patterns of use and to explore associations between usage and informed decision making.

## Method

### Design

We conducted an observational study of users of a web-based PSA test decision aid (Prosdex*).* Observations were performed on the web-log data generated by Web server software when participants accessed Prosdex. After viewing Prosdex, participants were also required to complete an online questionnaire.

### Participants and Setting

The study took place in the context of a randomized controlled trial of Prosdex [17], and participants were those men allocated to the intervention group asked to view Prosdex*.* Participants were recruited via their general practitioners in South Wales, United Kingdom, and were included if they were 50 to 75 years of age and had access to the Internet. They were excluded if they had previously had a PSA test or prostate cancer, had insufficient understanding of English, or were identified by their general practitioner as having learning disabilities, significant mental illness, serious ill health, or terminal illness. For full details of the study protocol see Evans et al [17].

### Intervention

Participants were asked to access and view an online study version of Prosdex without supervision in their own home or in a setting of their choice. After viewing the website, participants were automatically redirected to an online questionnaire that included questions to assess informed decision making (described below) and asked participants to select from predefined categories their age, ethnicity, marital status, and educational level.

**Figure 1:**
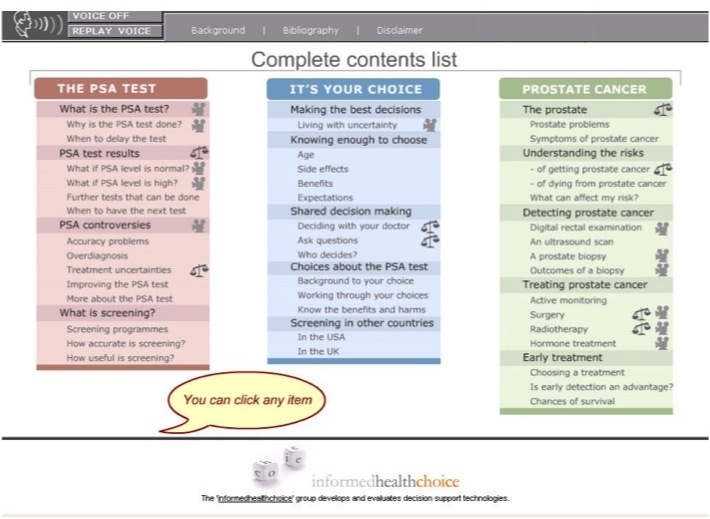
A screenshot of the Prosdex main contents list

**Figure 2:**
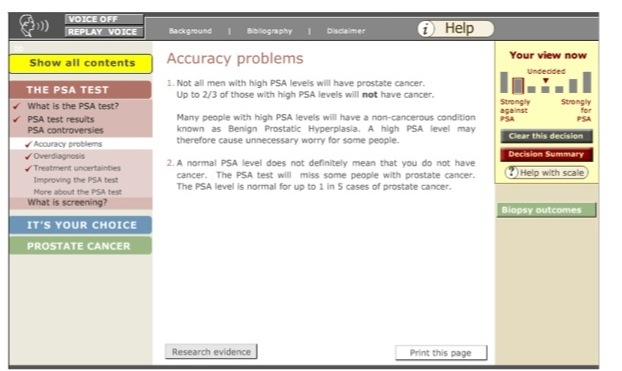
A screenshot of a page on Prosdex showing the decision stacker (right) and the navigation bar (left)

**Figure 3:**
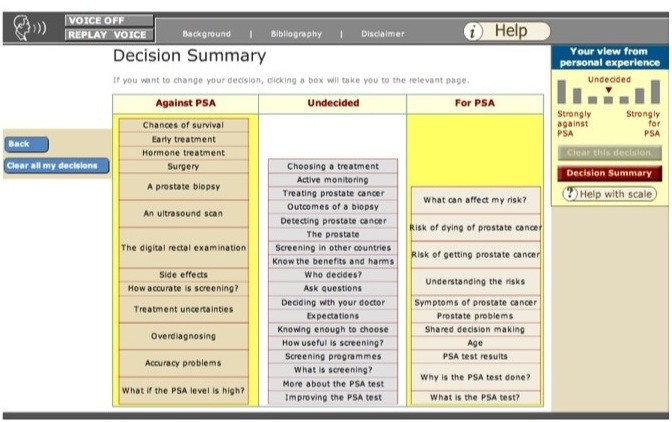
A screenshot of the decision summary in use

Prosdex is divided into three key modules: “The PSA Test,” “It’s your Choice,” and “Prostate Cancer” (Figure 1). Each module is further divided into sections and individual pages containing relevant information. Users navigate through the pages of the website using the navigation bar on the left-hand side of the screen (Figure 2). The website is an open learning environment, that is, users do not follow a fixed sequence through the site and are free to select the pages that are of interest to them. Users are also able to move freely between the different modules. When a page has been viewed, a tick appears in the navigation bar (see Figure 2). Each page contains links to other pages of related interest. The website is composed of text (accompanied by an optional audio track), images, video clips, and animations. Prosdex also contains a “decision stacker” (Figure 2) and “decision summary” (Figure 3). These features allow users to track and view their evolving decision status. The decision stacker is a five-point rating scale that allows user to rate whether they are for or against PSA testing at that point based on the information viewed in that section of the website. The ratings are recorded in the decision summary that users can view and print at anytime. Users are able to print each page by clicking a link at the bottom of the page.

### Outcomes

The key outcomes derived from the web-logs were:

- Total time spent on site
- Total number of main content pages accessed on site
- Time spent viewing each module (“The PSA Test,”’ “It’s your Choice,” “Prostate Cancer”)
- Time spent viewing each section (eg, “What is the PSA test?” and “PSA test results”
- Time spent viewing each page (eg, “Why is the PSA test done?”)
- Number of pages accessed in each module and section and actual pages accessed
- Total number of interactive elements used (ie, videos and animations)
- Whether the participant tracked their decision using the “Decision Stacker”
- Whether the participant used the integrated print function

The 3 components of informed decision making assessed were: (1) knowledge of PSA testing and prostate cancer-related issues; (2) attitudes toward testing; (3) behavior, for which a proxy measure was the declared intention to have the PSA test.

### Measurement of Outcomes

Prior to the web-logs being collected, the Prosdex website was coded so that each page/file request had a unique code. By doing this, we were able to determine from the web-logs the pages that had been viewed and which multimedia elements had been accessed by each participant.

The web-log files were generated by web-server software that tracked users’ interaction with the server*.* When a participant clicked the mouse button to view a particular page or interactive component of the website (eg, video), the browser sent a request to the web-server for a specific page or file. This created a log entry in a transfer log file, also known as a web-log. For each participant, a web-log was generated that contained detailed information about each request the server received from that participant’s web browser. Web-logs generated for Prosdex included information regarding the Internet Protocol address, time/date of each visit, the names of the pages/files requested, and the time difference in seconds between two consecutive page/file requests.

The component outcomes of informed decision making were measured in an online questionnaire that participants completed immediately after viewing Prosdex*.* Knowledge and attitude were assessed using sets of previously validated questions for PSA testing [18], and intention to undergo PSA testing was assessed using a single question which was answered on a Likert scale ranging from 1 meaning “definitely not” to 5 meaning “definitely will.”

The study received ethical approval from South East Wales Research Ethics Committee, 06/WSE04/13 (REC reference number). All participants gave informed consent before taking part in the trial after the nature and possible consequences of the study were explained.

### Analysis

Frequency distributions were made to assess participants’ use of the site (eg, total number of pages selected and total time spent on each page). Data were examined for outliers. To assess whether there were distinct subgroups of users characterized by the number of pages accessed, we examined the frequency distribution and the Q-Q (Quantile - Quantile) plot.

Correlations between the number of pages accessed and informed decision making outcomes (knowledge, attitude, intention) were assessed at the individual level using Spearman’s rho. For comparisons across subgroups, *t* tests and Mann-Whitney tests were employed. Significance level of alpha = .05 was chosen for all tests, and all statistical analyses were performed using SPSS version 12 (SPSS Inc, Chicago, IL, USA).

## Results

### Participant Sample

There were 129 participants (all men) allocated to the Prosdex intervention group in the associated RCT [17]. Of these, 82 successfully viewed Prosdex and had web-logs available for analysis. Web-logs were not available for 47 participants: 30 of the 47 did not attempt to access the site, and 17 attempted to access the site but then encountered software problems (eg, were unable to install Flash media player).

For 8 of the 82 men who successfully viewed Prosdex, we found an excessive amount of time spent viewing particular pages, indicating that these men had likely left the computer on for long periods of time with no interaction (in one case this was 11 days). Viewing times for these participants distorted the mean amount of time spent on each page, section, module, as well as the site as a whole, and were therefore excluded from descriptive analyses relating to time.

Of the 82 men whose web-logs indicated that they had successfully viewed Prosdex, 73 completed the online questionnaire and provided complete outcome data. Data for these 73 men were included in correlation analyses. The 17 men who attempted to access the Prosdex site but were unsuccessful (therefore providing no web-log data) had, however, completed the online questionnaire and provided complete outcome data. These men were compared with the 73 men who successfully accessed the site and who had complete outcome data.

### Participants’ Characteristics

Table 1 presents participant characteristics of the sample. Most men were between 50 and 59 years of age, married or living as married, and white. In addition, 36 out of 82 (44%) had a graduate or postgraduate qualification.

**Table 1:** Participants’ characteristics (n = 82)

Characteristic		Percent (Number)
**Age group**		
	50-59	61 (50)
	60-69	22 (18)
	70 or over	6 (5)
	Unknown	11 (9)
**Highest level of education**		
	No formal qualifications	11 (9)
	School leaver age 16 (formal qualification)	20 (16)
	School leaver age 18 (formal qualification)	8 (7)
	Clerical or commercial qualification	6 (5)
	Graduate or post-graduate qualification	44 (36)
	Unknown	11 (9)
**Marital status**		
	Married or cohabiting	81 (66)
	Single or never married	2 (2)
	Divorced or separated	6 (5)
	Unknown	11 (9)
**Ethnicity**		
	White	88 (72)
	Mixed race	1 (1)
	Unknown	11 (9)

### Prosdex Use

Table 2 presents a summary of the outcomes relating to participants’ use of Prosdex and the 3 modules: “The PSA Test,” “It’s Your Choice,” and “Prostate Cancer.” Table 3 presents a summary of the outcomes relating to participants’ use of the interactive features, including videos and animations.

**Table 2:** A summary of participants’ use of Prosdex: mean time (seconds); mean number of pages viewed; percentage of available pages viewed and percentage of videos/animations viewed among men in the low, intermediate, and high access groups

		Time		Access				
Module/Section		Mean (SD)^a^ Time in Seconds Spent in Module/ Section	Range	Total Number of Pages in Module/ Section	Mean Number (SD)^b^ of Pages Viewed	Percent of Available Pages Viewed Among Men in Each Group		
						Low Access (n = 37)	Intermediate Access (n = 18)	High Access (n = 27)
PROSDEX site		1191 (914)	75-3672	60	32 (21)	19	60	94
**The PSA Test**		412 (329)	0-1385	19	11 (6)	37	58	91
	Introduction	8 (19)	0-99	1	0.5 (0.5)	24	28	37
	What is the PSA test?	91 (66)	0-310	3	2 (1)	67	85	94
	PSA test results	132 (129)	0-772	5	3 (2)	45	68	92
	PSA controversies	103 (118)	0-420	6	3 (2)	28	44	93
	What is screening?	76 (87)	0-327	4	2 (2)	22	56	97
**It’s Your Choice**		218 (263)	0-996	19	9 (8)	5	51	97
	Introduction	13 (15)	0-77	1	1 (0.5)	54	83	93
	Making the best decisions	18 (49)	0 -394	2	1 (1)	4	58	94
	Knowing enough to choose	65 (85)	0-302	5	2 (2)	1	70	99
	Shared decision making	49 (70)	0-285	4	2 (2)	3	42	98
	Choices about the PSA test	44 (70)	0-341	4	2 (2)	2	32	99
	Screening in other countries	29 (42)	0-212	3	1 (1)	5	39	94
**Prostate Cancer**		428 (449)	0-1824	22	12 (9)	15	70	94
	Introduction	15 (14)	0-63	1	1 (0.5)	43	100	96
	The prostate	70 (68)	0-231	3	2 (1)	20	70	95
	Understanding the risks	60 (82)	0-503	4	2 (2)	12	76	100
	Detecting prostate cancer	114 (169)	0-733	5	3 (2)	17	63	91
	Treating prostate cancer	115 (180)	0-883	5	2 (2)	10	68	90
	Early treatment	50 (59)	0-228	4	2 (2)	12	65	94

^a^ based on 74 participants (8 outliers excluded)

^b^ based on 82 participants (8 outliers did not sufficiently distort usage data)

**Table 3:** A summary of participants’ use of video clips and animations on Prosdex among men in the low, intermediate, and high access group

Module/Section	Number of Videos (Animations) in Each Module/ Section	Percent of Available Videos (Animations) Viewed Among Men in Each Group		
		Low access (n = 37)	Intermediate Access (n = 18)	High Access (n = 27)
PROSDEX site	25 (8)	0.5 (2)	8 (16)	8 (21)
The PSA Test	9 (2)	1 (5)	3 (14)	2 (30)
It’s Your Choice	1 (2)	0 (0)	0 (6)	4 (13)
Prostate Cancer	14 (4)	0 (1)	12 (22)	12 (21)

Participants (n = 74, outliers excluded) spent a mean of 20 (SD 15) minutes on Prosdex. The shortest time spent on Prosdex was 1 minute and the longest time was 61 minutes. Participants spent a mean of 7 (SD 5) minutes on the “The PSA Test” module, a mean of 4 (SD 4) minutes on the “It’s your Choice” module, and a mean of 7 (SD 7) minutes on the “Prostate Cancer” module. The longest time spent on each module was 23 minutes, 17 minutes, and 30 minutes respectively. The relatively large standard deviations obtained highlight the large variability in the time that men spent on the modules.

The participants (n = 82) viewed a mean number of 32 (SD 21) out of a possible 60 main content pages on the Prosdex site with only seven men (8.5%) viewing all 60. A mean of 11 (SD 6) pages out of 19 were viewed from the “PSA Test” module. Eight men (10%) viewed all 19 pages while four men (5%) did not view any pages from this module. A mean number of nine (SD 8) pages out of 19 was viewed from the “It’s your Choice” module, with 17 men (20%) viewing all 19 pages, and 16 men (20%) not accessing any pages. A mean number of 12 (SD 9) pages out of 22 was viewed from the “Prostate Cancer” module. All 22 pages were viewed by 19 men (23%) while 13 men (16%) did not view any pages from this module. Overall, men spent longer and viewed more pages in the “PSA Test” and “Prostate Cancer” modules than they did in the “It’s Your Choice” module.

The interactive features of the site included videos and animations. The mean number of videos viewed was 1 out of 25, and the mean number of animations viewed was 1 out of 8. Of the 82 men, 64 (78%) did not view any video clips, and 44 (54%) did not view any animations. Of the 82 men, 37 (45%) used the “decision stacker,” designed to facilitate involvement in the decision making process. The majority of these men only used the stacker once, and therefore, usage was minimal. Only 3 men out of 82 (4%) used the integrated print functionality, printing only one item of information each.

### Analysis to Identify Subgroups of Users

By examining the frequency distribution and Q-Q (quantile-quantile) plot of the number of pages accessed, we identified a bimodal distribution. The frequency distribution of number of pages accessed suggested two modes, at ≤ 40% and ≥ 80% of the pages, with a relative dearth of intermediate values. Therefore, we defined three groups in terms of the number of pages participants accessed: low access was defined as 0 to 40% (ie, 0 to 24 pages), intermediate access as 41 to 79% (ie, 25 to 47 pages), and high access as 80 to 100% (ie, 48 to 60 pages).

Table 2 highlights the difference in overall Prosdex usage between the three groups and also demonstrates that the difference in usage was fairly consistent throughout each module and section of the website. Men in the low access group viewed a mean of 37% (7 out of 19 pages) of the “PSA Test” module, 5% (1 out of 19 pages) of the “It’s Your Choice” module, and 15% (3 out of 22 pages) of the “Prostate Cancer” module. On the other hand, men in the high access group viewed, on average, over 90% of the available pages in each module: 17 out of 19 pages of the “PSA Test” module, 18 out of 19 pages of the “It’s Your Choice” module, and 21 out of 22 pages of the “Prostate Cancer” module. Table 3 shows that the use of videos and animations was low in all three groups, although the high access group viewed a greater percentage of available videos and animations than the low access group.

### Correlations Between Usage and Components of Informed Decision Making

Correlations between usage (measured by number of pages accessed) and the measures of informed decision making were assessed (Spearman rank correlation [ρ], two-tailed). Focusing on number of pages as an indicator of usage allowed for the inclusion of participants with outliers on time data. There was a significant positive correlation between the total number of pages viewed and the overall knowledge score (ρ = 0.69, *P* < .001). In other words, the more pages a user accessed, the higher their knowledge score.

A negative correlation was found between the total number of pages viewed and attitude to screening (ρ = -0.52, *P* < .001). That is, the more pages the user accessed, the less favorable their attitude to the PSA test became. A negative correlation was also found between total number of pages viewed and intention to take the PSA test (ρ = -0.44, *P* < .001). In other words, the more pages the user accessed, the less likely their intention was to have the test.

### Comparisons Between Groups

Significant differences were demonstrated in knowledge scores, attitudes towards the test, and intention to have the test between men who accessed less than 40% of the website and men who accessed 80 to 100%. Specifically, *t* test results demonstrated that those in the high access group (80 to 100%) had significantly higher knowledge scores (t_56_ = 6.35, P < .001), and significantly less favorable attitude towards the PSA test (t_48_ = -4.51, *P* < .001). There was also a significant and inverse effect of number of pages viewed on intention to have the test when comparing the high access and low access groups (Mann-Whitney U = 211, *n*_1_ *=* 31*, n*_2_ *=* 26, *P* < .001, two-tailed).

On comparing participants with successful and unsuccessful access to Prosdex*,* there were significant differences. Specifically, *t* tests demonstrated that those who were successful had significantly higher knowledge scores (t_36_ = 4.59 *P* < .001) and significantly less favorable attitudes towards the PSA test (t_43_ = -2.44 *P* = .02). There was no significant difference between the groups on intention to have the PSA test (Mann-Whitney U = 585.5, *n*_1_ *=* 73*, n*_2_ *=* 17, *P* = .71, two-tailed)

## Discussion

### Summary of Findings

This web-log analysis of men using an online decision aid demonstrates a strong correlation between increased usage and increased knowledge, a less favorable attitude to the PSA test, and a congruent reduction in intention to take the test. We have found a significant dose-response relationship whereby informed decision making increases with increased usage of the website.

We found that men who used Prosdex spent a mean time of 20 minutes viewing the website before quitting. Therefore, while increased access is preferable due to the significant dose-response relationship, developers realistically have roughly 20 minutes in which to support an informed decision online. However, it is possible that users would spend even less time viewing such a site outside of the research context, so this time frame may be further limited. As predicted, we also found that men who used Prosdex did not comprise a homogeneous group, and access was not characterized along a continuum. Instead, the bimodal distribution of the data highlighted two distinct groups of users, characterized as low access and high access. The key findings of this study have important consequences for the future design of decision aids, as discussed at the end of the paper.

### Strengths and Weaknesses

This is the first study to analyze the web-logs of an online decision aid collected during an online study. We not only successfully identified patterns of usage, but also demonstrated correlations with outcome measures obtained from an associated randomized controlled trial. Specifically, we were able to identify associations between actual usage of a decision aid and components of informed decision making. If decision aids are designed to facilitate informed decision making, it is important that we understand the type of usage that leads to this.

Using a novel method of analyzing website log files, we have identified research limitations that require improvement. First, web-logs were unavailable for 47 of the men; 30 men did not attempt to access the site. This is a relatively high nonparticipation rate that could have an impact on the overall findings. There was evidence of software problems as 17 of the 47 participants were unsuccessful in their attempt to log in to the site. Future web-based research should ensure that software support is made available to users to minimize participant dropout associated with software downloads. The second weakness related to interpretation of the time data and the assumptions made from the web-logs. Although a long time spent on a particular page could indicate that the user took time to read it, it might also mean that the user left the computer and returned later. Given that the web-logs were collected in the home context, where it is likely that there are more interruptions, it is possible that the time data may be overestimated. Therefore, we cannot be sure that time spent on the site meant that the user was viewing the page for that time.

We recommend that interpretations of web-log data should be based primarily on the number of pages viewed rather than time spent. These two measures are highly correlated, but the former is more stable and robust. The correlations between usage and the outcome measures must also be interpreted with caution. While the dose-response correlations suggest that increased usage leads to more informed decision making, it might be the case, for example, that men with more doubts and less positive attitudes toward the test prior to viewing Prosdex were more likely to spend longer viewing the site.

### Comparison with Existing Literature

Molenaar’s [11] research on an interactive CD-Rom for breast cancer also used computer transaction log data for observational analysis, but they conducted an observational study where researchers were monitoring access. The mean time users spent viewing the decision aids was much longer with users spending over 1 hour viewing the information on the CD-Rom. In comparison, we found that participants’ spent a mean of 20 minutes viewing Prosdex. Unlike our study, participants were asked to view Molenaar’s decision aid in the presence of a nurse, which potentially encouraged participants to spend longer viewing the information than they would under natural conditions. As such, we believe the 20-minute time frame observed in our study is a more realistic and ecologically valid representation of use, and decision aid tools should be designed with this in mind.

However, we should note that the differences in time spent might also be a function of the population sampled. First, there is some evidence to suggest that women are generally more motivated to be involved in decisions about their health [19]. Second, PSA testing remains controversial as the test is limited by its sensitivity and specificity, and there is uncertainty relating to the natural history and management of prostate cancer. As such, there is often no imminent time frame in which a man would need to make a decision about whether or not to have the PSA test, unlike the decision a woman might be required to make between breast-conserving therapy and mastectomy, as was addressed by Molenaar [11]. It is therefore important to examine the use of decision aids for screening procedures that are more accurate and for disease treatment options where the natural history and management of the condition are better understood.

The study has expanded on existing research [11] by examining correlations between usage and outcome measures. This allowed us to examine patterns of usage that lead to informed decision making, which is what decision aids are designed to facilitate. We also examined patterns of usage, which we propose should inform the future design of decision aids. Previous studies have yet to address this functional use of web-log data.

### Conclusions and Implications for Research and Practice

There is evidence that Prosdex promotes informed decision making in men, and we highlight factors that should inform the future design of decision aids. First, for the population using Prosdex*,* 20 minutes seems to be a critical time window in which we can realistically expect information to be accessed. This finding is significant as there has been a recent trend towards developing more sophisticated decision aids that take longer to use, which could be seen as over engineering. We demonstrated, however, that participants did not use the interactive features, and that the window of opportunity for information transfer to support decision making is narrow. Second, users of decision aids are not a homogenous population: there are different types of users characterized by their level of interaction with the decision aid. Therefore, developers need to design tools that sufficiently support and facilitate informed decision making among the different types of users, and should move away from designing one intervention for all.

**Figure 4:**
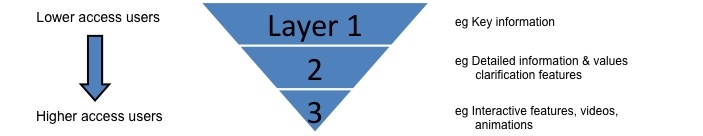
A diagram representing the stratum approach to developing decision aids

We caution against the simple response of developing shorter decision aids, with possibly two versions for high and low users. A more valid response, in our opinion, would be to move away from the traditional linear design of decision aids toward designing tools with a stratum approach (Figure 4), that is, decision aids with several layers.

The top layer would present users with the key messages and allow them to access the most important and relevant information with little navigation. This layer would be aimed at the low access group. It would ensure they receive the information they need to make an informed decision in a short time period. For those who wish to dig deeper, the more complex features (eg, interactive elements) and detailed information would be accessible. By adopting this stratum approach, developers could address the needs of different types of users and deliver the relevant and most important information within the relatively limited timeframe. We believe that developers who conduct further research and decision aid development along these lines will be able to support informed decision making for the greatest number of people. Additionally, the “golden” 20-minute time limit, found in the current study, could provide a useful heuristic for other developers. However, we recommend that web-log analysis be an integral part of the development process for online decision aids as well as a tool for posthoc analysis, so that developers can establish the “critical window” that is relevant to their own situation.

## Abbreviations

NHS: National Health Service
PCRMP: Prostate Cancer Risk Management Programme
PSA: prostate specific antigen
Q-Q plot: Quantile – Quantile plot
RCT: randomized controlled trial
Web-log: website transaction log file
